# “Christ, the Great Physician”: Chaplains in 19th-Century British Asylums

**DOI:** 10.1093/shm/hkaf070

**Published:** 2025-11-13

**Authors:** Ute Oswald

**Keywords:** chaplains, asylums, insanity, religion, therapeutic

## Abstract

This article explores the role of chaplains in 19th-century British asylums, focusing on the duties expected of them and the challenges they faced. These pivotal figures have so far remained largely under historians’ radar, which belies their importance in these institutions. It is argued here that they provided not only pastoral and spiritual care but that they also took an active interest in the education and entertainment of their flock. Examples are taken from a variety of asylums, including Hanwell, Colney Hatch, Littlemore, and Rainhill, where chaplains were particularly active and where reports and journals offer a rich source of as yet untapped information. This article will highlight the therapeutic potential assigned to their ministry and aims to raise the profile of these 19th century clergymen who worked in close collaboration with superintendents under often demanding conditions.

## Introduction

In 1893, the following article was published in the *Journal of Mental Science*, then the most important medical publication for those specializing in the treatment of insanity. It was part of the regular feature “The Asylum Chaplain's Column” and entitled “Religion, and its Influence upon the Insane”:

The principal work of the asylum chaplain is to preach and conduct the … worship in the institution, and, in his visits among the different wards, present the truths and consolations of religion to … the inmates.(...) This is not, indeed, his whole work. If he is worthy of his position he will ever seek to be the friend of the patients, sympathising with them in their troubles, talking with them on any matter in which they show an interest, and inviting their attention to such objects as may divert their thoughts from themselves.^1^

The author of this editorial was the Reverend Thomas Downie, chaplain of the Royal Edinburgh Asylum, Morningside, highlighting the many ways in which he and his brethren had to “minister to minds diseased.” It indicates the multi-facetted role of the 19th-century asylum chaplain, as he was tasked with providing more than just religious services for both patients and staff.

Whilst religion as a feature of 19th-century asylums has been widely acknowledged in asylum historiography, the role of individual chaplains, conversely, appears to have attracted scant attention.^2^ Studies of asylum staff tend to focus on superintendents, attendants or administrators, yet the work of asylum chaplains can reveal a side of institutional life which is often overlooked.^3^ This is surprising, given the extensive primary source material available; it ranges from annual reports and recently discovered chaplain's journals to specially dedicated treatises and articles in the 19th-century medical press. It also includes reflective institutional accounts by asylum chaplains themselves.^4^ Bearing in mind that these accounts served to reinforce the value of their position, this article will use them to put these pivotal figures centre stage, reveal how varied their role was and how their ministry was imbued with therapeutic benefits which have so far gone unacknowledged.

The role of the chaplain cannot be explored in isolation but must be situated within the context of 19th-century asylums, and the position of religion within them. As part of the so-called moral treatment regime, which had developed at the turn of the 19th century amongst the belief in the curability of insanity, religion, alongside work and recreation, played an important role in the daily asylum regime and its provision became enshrined in law.^5^ However, as asylums and asylum populations grew in size throughout the second half of the 19th century, providing individualized care, another original component of moral management, posed a challenge not just for physicians but also for chaplains; they thus had to find new ways of reaching their flock. This study will therefore also chart the origin and development of religious activities in 19th-century asylums, taking into consideration the organization of services by chaplains, and how they had to adapt these to the needs of a very different and growing congregation.

On the flipside, religion could be used to manage and control large numbers of patients and, like other aspects of moral treatment, was assigned a variety of benefits. It was seen to be “civilising” and comforting as well as enjoyable and therapeutic. The chaplain, therefore, much like the superintendent, was in a position of power. However, with cases of religious insanity on the rise, there was also concern that religion might prompt mental ill health, and thus its role in asylums was subject to heated debates. This article will address this tension and show how, despite no real consensus on the matter, religion remained an important part of the asylum routine, and how chaplains were accrediting it with patient recovery and, in consequence, affirming their own role as “therapists.”

Within the 19th-century asylum and the regime of moral treatment, the chaplain was thus a figure of authority to an extent which has so far been undervalued. With this authority came expectations and pressures, and clergymen expressed these challenges in publications and reports. It took a special character to tolerate working in often isolated institutions and administering to patients who were not necessary all church-goers and frequently suffering from debilitating mental ill-health. In addition, chaplains were tasked with the provision of far more than spiritual care, as their duties also extended into the recreational sphere. This article will discuss these demands, and the skills chaplains were expected to possess.

Moral treatment in British asylums has been extensively discussed by historians of psychiatry, with more recent studies on recreation by Cherry and Munting, Ellis, Foster, and Golding adding important scholarship to it.^6^ Yet, religion, and the link between religion and recreation, has not.^7^ This is largely due to the lack of attention paid to the chaplain. Whilst superintendents are usually credited with providing amusements and attendants held responsible for implementing them, the recreational activities arranged by the chaplain remain mostly obscured from view. This article will therefore reveal how instrumental he was not just as “Father-Confessor,” but also as “Librarian-Keeper,” educator and entertainer, firmly cementing his position within the moral treatment regime. As he was often second only in remuneration and standing to the superintendent, it will also interrogate this special relationship, how both navigated potential tensions between the religious and the medical sphere and, ultimately, how chaplains slotted into the hierarchical and therapeutic asylum routine.

Voices of asylum patients are not always easy to locate and raise concerns of reliability and bias. However, this article will conclude by presenting several patient testimonies, which express the value they affixed to religion and their gratitude to individual chaplains. They reinforce the picture which emerged in the previous sections of a man who made a real difference to the institutional life of those who suffered from insanity and invite us to reappraise the 19th-century asylum, which so often has been depicted as a site of repression and doom, “where many patients were simply left to rot.”^8^ It also provokes us to consider the potential of religion and the clergy in the treatment or prevention of mental ill health today, providing a historical perspective to recent research, which found that religious people report to be the happiest.^9^ After all, as psychiatrist Paul Summergrad suggests, “spirituality and religious experience are too central to human experience and culture for mental health professionals or other clinicians to ignore.”^10^

Although religious life was also flourishing in private madhouses, here records are not as accessible; therefore, the focus of this study will be on chaplains working in county and charitable British institutions such as Hanwell Asylum, Rainhill Asylum, Littlemore Asylum, and Bethlem Hospital. Charitable asylums mostly catered for middle-class patients whilst county asylums were established for the pauper class, and in both, chaplains were particularly active and source material rich. Furthermore, it must be acknowledged that patients of different faiths were also increasingly accommodated, as Rob Ellis and Leonard Smith have shown.^11^ Often, Jewish patients would be visited by a Rabbi and Catholic patients had the right to see a priest.^12^ However, this study explores the Anglican chaplains, as the vast majority of the British asylum population were members of this denomination and because this is the predominant type of chaplain documented in the sources and historiography.^13^

## Religious services in 19th-century asylums

The connection between religion and the treatment of madness is longstanding. The first recorded Lunatic Asylum, Bethlem Hospital, was originally a Priory, built in 1247 with “religious and crusading zeal,” before focusing on the care of the sick and the mad from the fifteenth century onwards.^14^ One of the pioneering institutions for the insane, The Retreat in York, established in 1794 by the Quaker William Tuke, was conducted upon religious foundations with the so-called moral treatment at its core.^15^ This treatment was, as Anne Digby argued, “not so much a specific technique, as a range of non-medical treatments designed to involve the patient actively in his recovery.”^16^ Broadly speaking, it focused on kindness, an individualized relationship between doctors and patients, patient occupation, and religion.

Alongside work and recreation, as Leonard Smith has observed, “worship became an essential part” of the 19th-century asylum routine.^17^ This was not surprising, given the importance of religion in everyday life. As Gerald Parsons claimed, “Victorian Britain was (…) a profoundly religious society.”^18^ The Anglican Church was the Established Church in England and Wales, even if its definition is a “subject of considerable controversy.”^19^ It is impossible to discuss the diversity of denominations and individual religious experiences here, but it should be stressed that Evangelicalism was, as Dominic Erdozain suggested, “the movement that did most to define the modern religious landscape” because “it generated (…) Sunday schools, missions, popular hymns, and the moral seriousness that became the ‘common sense’ of the British in the nineteenth century.”^20^ Evangelicals were driven by “human sympathy as well as divine command,” founding a vast number of societies and leading the way in social reform.^21^ In fact, Anthony Ashley Cooper, the Seventh Earl of Shaftesbury and a major stakeholder in the asylum reform movement, was staunchly evangelical and an ardent supporter of the Sabbatarian movement.^22^ Consequentially, the provision of religious services became of increasing importance to those in charge of the insane and thus incorporated into lunacy legislation. The 1828 Amending County Asylums Act stipulated that the Justices of the Peace should appoint a salaried chaplain, licenced by the Bishop, for every County Asylum to “perform on each Sunday, and on the great Festivals, the Divine Services of our Church.”^23^ The 1845 County Asylums Act, which demanded the mandatory erection of asylums in each county, repeated the compulsory appointment of a chaplain for each asylum, but now it was the Committee of Visitors who were to select and, if warranted, remove him, and who were also entitled to suggest holding services at other times in addition to Sundays, Christmas Day and Good Friday.^24^

The movement to incorporate religious activities into the daily and weekly asylum routine gained momentum from the mid-19th century onwards. Whilst the 1847 annual report of the Commissioners in Lunacy, who were responsible for overseeing the provision of asylum care, did not mention chapel, chaplain or divine service at all, from 1853 onwards, these terms figured increasingly.^25^ From 1890, Divine Service had its own section within each report, alongside amusements and occupation, thus situating it firmly within the main elements of moral treatment. Superintendents included accounts of religious life in their institution, and, especially from the middle of the century onwards, chaplains were given the opportunity to contribute their own reports. Within these, they outlined their accomplishments and challenges during the year, and, on rare occasions where more extensive chaplain's journals have survived, we can see their daily records.^26^

One reason for this increase in documentation no doubt lies in the general directives of the County Asylums Act of 1845, which demanded more detailed reports of asylum life and patient records. Another perhaps in the fact that the number of county asylums increased considerably between 1847 and 1890; in 1850, there were 24, by 1870 this had risen to 50, and by 1900 there were 77 such institutions.^27^ Asylum populations also grew during this period. For example, the average number of patients in asylums on January 1, 1827 was 116, rising to 387 by 1860 and 802 by 1890.^28^ However, the actual number of patients varied greatly; whilst in 1874 some provincial asylums, such as Littlemore Asylum in Oxford, catered for around 500 patients, others, such as Hanwell Asylum or Colney Hatch Asylum, accommodated three to four times as many.^29^ This is important to bear in mind when considering the implications of providing services and pastoral care.

Chaplains were responsible for Sunday and festive services as well as burials, baptisms, and confirmations. Asylum records show that mid-week services were added in some institutions to the usual services on Sundays, and that all appear to have been popular. At the North and East Ridings County Lunatic Asylum in York, which in 1859 treated 445 patients, the chapel could accommodate 300 and was reported by the Lunacy Commissioners as “filled” on Sundays for two services performed by a chaplain who also visited on Wednesdays for a service and Fridays for spiritual aid.^30^ At Hanwell, religious services played a central role. In the 1839 annual report, its superintendent John Conolly explained that services were held at 11 a.m. and 3.30 p.m. every Sunday, which each lasted one hour, with staff in attendance as well as circa 200 patients.^31^ By 1842, the Sunday congregation had increased to 300 out of 953, whilst between 150 and 200 patients also attended recently introduced daily morning and evening prayers, each lasting around 15 min.^32^ After a new and bigger chapel was built in 1855, even more patients participated in services.^33^ However, here as well as at other asylums, the attendance at midweek services was hampered by patients' work commitments. They complained to the visiting commissioners and these in return expressed their regret, stating that work should not interfere with Chapel.^34^ Subsequent attendance figures seem to show that this had not been heeded; in 1886, 460 out of 748 male and 650 out of 1138 female patients were present at Sunday services, whilst for daily services, the figures stood at 186 and 150, respectively.^35^ At Brookwood, the second Surrey County Lunatic Asylum, the chaplain had, quite shrewdly, decided to hold weekday services in the workshop rather than the chapel; one way of facilitating, but possibly also limiting, participation, depending on the size of the workshop.^36^ At the Worcester Asylum, the chaplain Alfred Bond intended to change his daily visits from the wards to the laundry and the workshops, again, presumably to reach individually those who were working.^37^

The timing and frequency of services seemed not too dissimilar to those outside of the asylum, which in the morning were around 10 a.m., in the afternoon at 2 p.m. or 2.30 p.m., or in the evening anytime between 4 p.m. and 7 p.m.^38^ The length of services in the parishes depended on the length of the sermons, which could vary. In the asylum setting, however, it was necessary to keep services relatively short.^39^ Sunday services were always reported to be one hour, and weekday services between 15 and 20 min.^40^ Yet, not only the length of services had to be adapted to suit the recipients but also the type of language and topics employed. An article in the *Lancaster Gazette* demanded that the sermon during an asylum service “should be in the plainest language, free from every thing [*sic*] of an argumentative or doctrinal cast,” and that it should “inspire hope, and alleviate despondency.”^41^ John Conolly stipulated that the service was to be “so varied as to obviate fatigue or restlessness,” and that “some passages in the Scriptures, and some kinds of sermons, being particularly likely to be misapplied, (…) must be abstained from in reading and preaching.”^42^ These adjustments, in particular of content and delivery, were in response to concerns of religious insanity, igniting debates as to both the value and risk of providing religious services to patients in asylums.

## Wider benefits of religion and the question of religious insanity

Demonic possession had long been associated with madness.^43^ In the sixteenth century, Robert Burton saw religious insanity as “ensnarement by the devil”; yet, it could also be understood as a punishment from God.^44^ This interplay between Divine and Satanic Madness continued into the eighteenth century and was propounded by John Wesley. In fact, Wesleyan Methodism was predominantly held responsible for the so-called “religious enthusiasm” which could cause insanity, prompting physicians such as James Cowles Prichard to hold this “severe (…) and almost imprecatory style of preaching” responsible for “enforcing the terrors rather than (…) hopes and consolations” of religion.^45^

Yet, also outside of Methodist “ranting,” religious insanity more generally was increasingly charted in annual reports, usually under “religious excitement.”^46^ “Over-religiosity” became a topic much discussed amongst physicians such as John Conolly, who used case studies to illustrate the inward and outward symptoms of religious melancholy and religious mania in the *Medical Times and Gazette*, or Daniel Hack Tuke, who referred to it on numerous occasions in his *Dictionary of Psychological Medicine.*^47^ Yet, it also featured increasingly in the non-medical press, indicating that it was clearly of interest to the general public, too.^48^

Thus, the laws demanding the provision of religion in asylums were not unanimously welcomed by physicians. However, opinions varied widely and no real consensus could be reached. Some, such as the Bethlem physicians George Tuthill and Edward Thomas Monro were reported to have been “frankly hostile” to the provision of religious services, and the Bethlem chaplain, Reverend O’Donoghue, admitted that as “paradoxical as it may seem, certain forms of insanity are nourished by the reading of the Bible or by attendance at a religious service.”^49^ Yet, the extent to which “overstretched (…) religious views may have been a first symptom rather than the cause of insanity” was also debated, alongside potential predispositions to the disease. For example, Dr Rooke Ley, Superintendent at Prestwich Asylum, asserted that “religion by itself is incapable of upsetting a sound mind,” but that “revivalism, spiritualism and mesmerism (…) do act in upsetting the balance of a mind already weakened by disease or by inherited taint.”^50^

It was therefore no surprise that chaplains commented on the very different approaches required in comparison to those their peers employed outside the asylum. In the article “The Good Asylum Chaplain,” published anonymously in the *Journal of Mental Science* in 1893, it is outlined how “in his sermons [he] remembereth the peculiar class of persons whom he addresses, and escheweth all theological disputations; he is simple in his language, loathes affectation, is earnest in the manner and consolatory in the matter of his discourse.”^51^ However, in a clear signal of the varied needs of this special congregation, it also advised that “simple and uncontroversial preaching is (…) not to be confounded with monotonous platitudes, which are not only a poor compliment to the more intelligent patients, but are intolerable to that portion of the auditory which consists of the staff of the asylum.”^52^ Reverend Edwin Pulling at Littlemore Asylum was acutely aware of this and adjusted his style to ensure that “the medicine we administer to our Patients is of a mild and healing description.”^53^ His choice of words is poignant; it highlights the therapeutic value placed on religion and its potential parity with medical intervention.

In fact, those who were in favor of religious services for the insane stressed the many benefits these afforded, which the chaplain could take some credit for. They ranged from “remedial usefulness” and “enjoyment” to “comforting” and “subduing,” indicating both therapeutical and managerial advantages and placing religion firmly within the realm of moral treatment.^54^ Colney Hatch chaplain Henry Murray applauded the “moral advantage gained” by attendance at Sunday services, and J.T. Burt, chaplain at Hanwell, referred to the importance of religious texts to inculcate “truths (…) adapted to alleviate mental distress and remedy the disorder of the moral constitution.”^55^ Services could also provide a break in the monotony of asylum life, and remind patients of their lives prior to admission, if they then had been so inclined. Additionally, behaviour at services was used as a barometer of recovery, as here patients could demonstrate “decorum” and self-control; much needed attributes of life outside the asylum walls.^56^ For example, at Wandsworth Asylum, chaplain Richard Greaves praised his congregation, which “considering the malady wherewith most who compose it are affected, has been remarkably orderly and attentive.”^57^

This focus on order and decorum during services within the asylum chimes with the social control aspect of moral treatment and is reflected in several treatises and reports.^58^ The highly respected superintendent at the Crichton Royal Institution in Dumfries, W.A.F. Browne, observed that “the effects in each individual are probably as different as in the members of an ordinary congregation, but the general impression produced is that of reverence and order” as each “meeting is almost invariably distinguished by perfect decorum and propriety.”^59^ The Commissioners in Lunacy also commented on how patient behaviour at the services at Rainhill County Lunatic Asylum was “most orderly in conduct and demeanour,” giving an official stamp of approval to the way the chaplain controlled his flock.^60^

Browne further noted that “religious service has a wonderful effect in subduing their irritation, in calming their minds and composing their spirits, not only during the time of the service, but during the remaining hours of the day of rest.”^61^ Reverend Pulling confirmed this, commenting how “the good effects resulting from these religious services must be felt throughout the whole Establishment.”^62^ This seems to indicate that patients could be kept quiet, or “managed,” for a longer period of time following religious services, which must have been a welcome development for those having to look after them and reinforces the power the chaplain possessed. Yet, Pulling also referred to the curative effect of paying “the greatest attention to (…) religious duties,” which has benefitted patients recently discharged as recovered as well as those deemed incurable.^63^ Here then, another direct reference to the healing potential not just of religion, but of those facilitating it: the 19th-century asylum chaplain. However, it would be wrong to reduce the work of the chaplain to the provision of religious services. Of course, they comprised one of his main duties; yet he accomplished far more than this.

## The 19th-century asylum chaplain: expectations, challenges, and pressures

As mentioned above, the County Asylums Act of 1828 had stipulated the appointment of a chaplain to minister to the insane, and asylum annual reports increasingly contained a section written by the clergyman in charge. What transpires from these “Chaplain's Reports” is the multi-faceted role which saw him not only “preach and conduct the other exercises of worship” but also “seek to be the friend of the patients,” to be a “mediator between them and the superintendent” as well as act as a “Librarian-keeper.”^64^ At Hanwell, a set of rules and regulations required that the chaplain, apart from conducting services, “should daily visit and superintend any Schools or places of Instruction existing in the establishment,” should keep a daily journal, be in charge of the library on both sides, and confer with the committee with regards to a suitable selection of reading material.^65^ At Holloway Sanatorium, a charitable asylum for the middle and upper middle classes, when the position of a permanent chaplain was to be filled, the search was for “a man of broad and liberal views, of some musical ability, genial in manner, and earnest in his work, one who can gain the confidence and respect of patients and staff.”^66^

This was no easy task. Several medical publications engaged with the expectations levied at and characteristics desired of a good asylum chaplain. After all, as Anna Shepherd asserted, “spiritual guidance was seen as necessary for both staff and patients, and a good clergyman ensured a well-attended asylum chapel, which pleased the Commissioners in Lunacy.”^67^ The challenges of “ministering to the mind diseased” were acknowledged in the *Journal of Mental Science*, affirming that “*the acquisition of the necessary knowledge is not very easy, and the mode of intercourse with the various patients is not to be learned off-hand”*.^68^ The chaplain was supposed to “make it his daily study to fathom the psychical conditions of his afflicted flock”, and at Bethlem, Reverend O’Donoghue asserted that “the chaplain must find more than half his work outside the chapel” as “father-confessor.”^69^ He outlined further duties as taking some of the patients “under proper escort to see some of the sights, or for a walk in the suburbs and country,” writing up the magazine, or sending “a chatty letter (…) to an old patient.”^70^ He also suggested that “he may be partly a charity organisation inquiry officer and partly a well-equipped almoner in some Samaritan scheme for the present care of a patient's family and the after-care of convalescent patients.”^71^ This indicated a varied pastoral role, and underlined the important position chaplains held within the asylums. Yet as institutions grew, this undoubtedly became more difficult, and O’Donoghue acknowledged that the congregation was “perpetually changing—seldom for the better” as, at Bethlem, recovering patients had been “drafted to a comfortable house,” replaced by “patients not so far advanced on the road to health.”^72^ Individualized pastoral attention thus had its challenges.

Another, earlier, indication of this can be detected in the 1850 report by Robert F. Chambers, chaplain at the Surrey County Lunatic Asylum at Brookwood, in which he “rejoice[d]” to proclaim that “with very few exceptions” patients “welcome my visits among them as those of a friend.”^73^ As his time was “no doubt, much more occupied by the very enlarged number of the patients in respect of my visits throughout the ward,” he was grateful that this had been acknowledged by the Visiting Justices and that his salary was increased as a result.^74^

Perhaps, the stigma attached to working in an asylum and issues surrounding accommodation discouraged suitable clergymen, too. Reverend P.T. Syrée, chaplain at the Kent Lunatic Asylum, Chartham, complained that “the outer world have yet to learn their Christian duty towards the insane” which applied also to “the Church, who appear to shun the very approach of the insane, as though insanity and leprosy were akin!”^75^ Furthermore, as not all chaplains were given housing, another disadvantage for non-resident chaplains was the fact that they had to find a residence in reasonable proximity, because Divine Services were often early in the morning. Reverend William Iago of the Cornwall Asylum at Bodmin had expressed his struggles in this regard, and John Conolly had acknowledged this problem at Hanwell by suggesting that morning services were moved back an hour, which would “be more convenient to the chaplain, who is not usually a resident officer in asylums.”^76^

The pressures of the post were thus keenly felt. Edwin Pulling commented in 1855 that the “daily intercourse with the mentally diseased was, at times, very irksome and spirit-depressing,” but that he received “encouragement from the Lord.”^77^ Although there is evidence in reports and journals that some chaplains were upholding relationships with their peers, especially as replacements during holiday or sickness, there does not appear to be the same level of knowledge exchange and network creation as was the case for physicians.^78^ The “Asylum Chaplain's Column” in the *Journal of Mental Science* was therefore an important platform on which information could be shared. Reverend Henry Hawkins, chaplain at Colney Hatch, pointed out in his support for this column that “We chaplains are in a position of partial isolation, living at a distance from one another, and rarely meeting,” stressing that “the duties of the parochial clergy, our neighbours, differ in many respects from our own, and their experiences do not qualify them to supply the particular advice and counsels of which we may sometimes stand in need.”^79^ Hawkins radiated passion and commitment in the treatment of the insane and wrote several articles and tracts with regards to the challenges not just for asylum chaplains but also for asylum attendants.^80^ A reviewer of one of his publications praised Hawkins’ efforts and his caring attitude towards patients not only during their stay in the asylum but also on discharge. Hawkins founded “The After-care Association for Poor and Friendless Female Convalescents on Leaving Asylums for the Insane” in 1879, later known as the Mental After Care Association (MACA), which aimed to provide work and accommodation “to help the recovering patient safely bridge the gap between the therapeutic asylum and full participation in social and economic life, without sinking into the social residuum.”^81^ This is clear evidence of the legacy of one particular asylum chaplain's work, which, again, is often overlooked.

## The chaplain as entertainer and educator

As the previous section has indicated, asylum chaplains were not just providing services and pastoral care on the wards. Several of them proved that “their heart and soul are real and truly in the work” and performed duties, which were perhaps outside those expected by an asylum clergyman. For example, Henry Hugh Higgins, who served from 1853 until 1893 as chaplain at Rainhill Asylum, was particularly invested in the instruction and entertainment, religious and otherwise, of his charges. In 1853, he held “a course of lectures on the East (…) illustrated by dissolving views of Oriental Scenes, showed by the aid of the Magic Lantern’, from which much gratification and pleasant instruction was derived.”^82^ As often the case, there was a correlation between the personal interests of the organizer and the activities provided. In addition to his “proper treatment of insane people,” Henry Higgins had “an intense love of natural history,” (…) he was a keen and skilful sportsman, and as a companion in a fishing expedition, he was charming’. Furthermore, “he was also an excellent musician, and in fact a good ‘all round’ man.”^83^

Higgins’ profile illustrates the wide range of activities offered by asylum chaplains; the Magic Lantern Shows were a popular pastime also at Littlemore Asylum. Here, Reverend Pulling introduced these shows into the separate wards during the Winter months, reporting that patients were “highly delighted with this hour's amusement.”^84^ Interestingly, he also stated that although his duties were essentially religious, these presentations were not “inconsistent with his sacred calling, to endeavour to cheer the melancholy, and amuse the more happy disposed portion of his flock, by entertaining them with some innocent recreation.”^85^

The Magic Lantern shows continued through the 1860s at Littlemore, with images taken from Natural History or humorous figures for between 50 and 100 patients.^86^ It had previously been considered dangerous as it took place in a dark room but “it is highly amusing to hear the droll remarks in response” and “nothing of any unpleasant nature has ever happened.” Even when it ceased to be a novelty for some, the new patients were excited by it and the chaplain found it “a great source of amusement to the more recently admitted ones, some of whom never saw anything of the kind before or since they were children at school.”^87^ In 1863 the chaplain introduced—perhaps surprisingly, given the bizarre nature of this show—a Phantasmagoria Lantern twice a month; once on the male side and once on the female.^88^

Although the Magic Lantern Shows seemed incongruent with the official duties of a chaplain, often their content was educational. As mentioned above, slides of Natural History or Geography formed part of the programme, in a nod to the role of the chaplain as educator. This was in line with developments in wider society, as the YMCA was established and Working Men Clubs, Mutual Improvement Societies and Sunday Schools proliferated.^89^ Most of these were church initiatives, for example the Working Man's Mutual Improvement and Recreation Society founded by clergyman Henry Solly in Lancaster in 1860, and others involved superintendents, for example John Conolly, who was an active member of the Society for the Diffusion of Useful Knowledge.^90^ As part of the so-called “rational recreation” movement from the middle of the 19th century onwards, Peter Bailey confirmed that “the recreations which recommended themselves to respectable tastes were those with some manifest moral or improving content,” such as lectures, certain literature and general instruction.^91^ Especially Sunday Schools “contributed both to the spread of literacy in the working classes and to the creation of a ‘respectable’ working-class culture,” but weekday elementary education was also often based on religious doctrine.^92^ It is therefore perhaps no surprise that chaplains took it upon themselves to transpose this into the asylum setting.

Steady mental pursuit had been deemed beneficial by several physicians.^93^ Chaplains, too, praised the positive impact of education. In his support for the establishment of a specialist platform to exchange experiences, later to become the “Asylum Chaplain's Column” in the *Journal of Mental Science*, Henry Hawkins listed as one of the areas for discussion “the formation and management of classes.”^94^ This demonstrates the importance placed upon them, and they were in most cases well-structured, with subjects ranging from Reading and Writing to Geography and Arithmetic. Sometimes asylum staff delivered the lessons, but often specialist schoolmasters and schoolmistresses were employed. At Rainhill Asylum, Reverend Higgins had purchased large maps of the Eastern and Western Hemispheres which were put up in the library and used for weekly classes in Geography for female patients. Higgins’ special enthusiasm for education was not surprising; in addition to being a renowned botanist, he was also inspector of the National Schools in Liverpool from 1842 to 1848.^95^

Many asylums offered classes which received special mention in chaplain's reports, interestingly alongside other “games and pastimes,” indicating that chaplains kept a close eye on other recreational activities too.^96^ Hanwell had a particularly impressive educational programme. When John Conolly took over as superintendent in 1839, he included in his annual reports tables charting the educational standard of the patients.^97^ Conolly stated that “amongst those ‘unable to read’ are some who would be teachable.” This was especially applicable to female patients, but he observed that some male patients were also “very eager to learn” and he thus recommended the establishment of schools in the asylum.^98^ In 1842 this was realized, and the chaplain, J.T. Burt, described the development of his classes in quite some detail in his reports. In this first year, thirty of the more intelligent patients were selected to take part, and classes of six or eight “assembled by themselves.”^99^ Although Burt confessed to being apprehensive at first, he found that reading became very popular, and thus the classes increased in number.^100^ Classes took place in dayrooms of respective wards, with seventy to eighty patients, and the chaplain, “keen to instruct the ABC and spelling to those currently illiterate,” was “anxious to extend the measure as generally as possible.”^101^ Interestingly, the report also mentioned that patients actively requested instruction in Reading and Writing, which indicates a certain level of agency.^102^

The report of the Visiting Justices the following year favorably mentioned the lessons for patients.^103^ As suggested by the chaplain and the resident physician, a schoolmaster and schoolmistress were now employed for a time for patients who were “thought capable of deriving any benefit from it.”^104^ However, despite the seeming popularity, tuition was abandoned a few years later. Conolly, expressed his “deepest concern” when he “unexpectedly found the schools closed, by order of the visiting justices” but conceded that “it was scarcely extended to the most intelligent of the patients; so that the difficulties to be met by the schoolmaster and schoolmistress were very great.”^105^ Even though in the end at Hanwell these classes were discontinued, overall, it was remarkable to what extent education was part of asylum life, and in turn, how chaplains were instrumental in introducing educational activities into these institutions. It is yet another aspect of the chaplains’ work which has gone largely unrecognised.^106^

## The chaplain as librarian

Invariably, a main constituent of these classes were the reading materials. It fell within the remit of the chaplain to provide a range of books and periodicals and to establish, wherever possible, an asylum library.^107^ At Littlemore, the chaplain's report regularly mentioned the “small library of books” and how it engendered great enjoyment for the patients.^108^ It was a lending library, and as such afforded patients the freedom and agency to take advantage of this recreational source. Interestingly, populist publications, such as the *Illustrated London News* and *Punch*, formed part of the range, as well as newspapers which patients received from their friends.^109^ This demonstrated a certain tolerance towards non-religious texts and interactions with the outside world, as well as a certain level of literacy. Reading had become one of the most popular pastimes and the custom of reading aloud also took hold, partially thanks to the establishment of Mudie's circulating library in 1842 and of Penny Readings.^110^ As Pamela Horn observed, the quantity of printed material “grew with astonishing speed, stimulated by rising consumer incomes, greater leisure and improved literacy.”^111^

This was reflected in the asylum population, at least at Hanwell, where, in the year 1843, out of 180 admitted patients, 11 were classed as well educated, 104 could read and write and 33 could just read.^112^ It was therefore no surprise that in 1857 the chaplain John May circulated for Sunday reading certain “eagerly sought for” periodicals, like the *Cottager* and the *Sunday at Home*, which were “inculcating the best principles,” yet were equally amusing and interesting.^113^ By 1871 an impressive selection of six daily, 51 weekly and 22 monthly journals were at the patients’ disposal, which included *The Telegraph, The Athenaeum, Fun, The London Journal*, *The Art Journal* and *Cassell's* alongside the more common religious publications such as *Leisure Hour*, *Good Words* and the *Cornhill Magazine*.^114^

Religious texts were obviously a core part of the asylum literary stock. As Gerald Parsons stated, “the vitality and passion of Victorian religious life” was “reflected in this sheer quantity and variety of religious literature produced by the various churches and religious societies,” and hence there was no shortage of publications to choose from.^115^ Whilst religious periodicals were consumed for leisure purposes, the Bible as well as Prayer and Hymn books were used during asylum services and specially established Bible classes. The latter were again a reflection of practices in wider 19th-century society. In addition to individual devotional sessions, Julie Melnyk described the popularity of so-called “cottage meetings,” which were gatherings in parishioners’ houses for Bible study and prayer.^116^ At Hanwell, Reverend May reported that he was pleased about the popularity of his Bible lectures during the Winter, which were held in the evenings for both sexes and “attended by about 130 of the more intelligent Patients.”^117^ Interesting is the fact that a differentiation of participants according to state of mind and education was often made. On a similar note, at Littlemore, the chaplain noticed that the more rational and convalescents would like to have bibles of their own.^118^

In fact, the benefits of prayer books to staff were often lauded. David Uwins, for example, claimed that “perhaps the minds of the attendants, whose routine of duty is calculated to foster anyt-hing *[sic]* but religious impressions, may … be preserved from complete obliteration of such impressions, by the mere formality of even prayer-reading.”^119^ Clearly, prayer, through its routine application and meditative repetitiveness, was imbued with therapeutic potential, but not just for staff.^120^ Thomas Downie argued that the “Prayer Book has a soothing tendency very beneficial to sorrowful and disturbed spirits’ and physician Arthur Ladbroke Wigan described how “the repetition of these [prayers]” was “soothing (...) and tranquillises the morbid emotions of the brain.”^121^

## The chaplain and music

This soothing effect was also ascribed to religious music and, alongside patients and staff, chaplains were involved in its organisation.^122^ Colney Hatch chaplain Henry Murray recounted that the choral service “tends to soothe and quiet many,” even the most talkative, once the organ struck up.^123^ Hymn books formed a central part of religious musical activity, and although congregational hymn singing had originated in Wesleyan Methodism, Anglican Evangelicals soon adopted this practice as part of their worship.^124^ Hawkins proposed that services “should be made bright with simple chants and hymns in which the congregation can join,” discussing the possible “composition of the *choir* (…), whether consisting of members of the staff, male or female, or of patients, or of both associated, whether assisted in some cases by friends outside.”^125^ This, he posited, might result in “the improvement of asylum choirs, which, in many cases, contribute greatly to the devotion and attractiveness of the services.”^126^

Several annual reports indeed showcased the involvement of both patients and staff. At the Devon County Lunatic Asylum for example, the chaplain reported that the choir “is often composed of patients” and that “the interest taken by the head attendant, Mr Headon, and also some of the other attendants in assisting to improve the musical part of our services on Sundays, has been attended with great success, and is much appreciated by my congregation.”^127^ At Warneford, although “a very excellent paid choir” performed on Sundays, patients still attended choir practices during the Winter months, and at the Dorset County Lunatic Asylum the chaplain stated that “the Sacred Singing Classes on Wednesday and Thursday evenings have been carried on as usual, and I have great pleasure in stating that the musical portion of the Patients and Attendants have taken great pains to render their part in our services with much credit to themselves.”^128^

Much importance was placed on the musical accompaniments to the choir and congregational singing. The organ is mentioned frequently.^129^ At Hanwell, William Ellis commented that the profits from the Bazaar would probably be used for the purchase of an organ, so the “patients will feel proud of it,” highlighting the special, perhaps “sacred,” function this instrument represented; he also expressed the wish that it may be played by a patient.^130^ This was a recurring theme. At Devon, “for many years a patient who had been an accomplished musician, was Organist,” and at Littlemore the chaplain had purchased a harmonium for their chapel where “a Female Patient, who was very musical, performs on it.”^131^ At Colney Hatch, a female patient, who usually played the piano, acted as organist, but as she was sometimes too ill, the chaplain was waiting for a professional to replace her.^132^ Here perhaps an indication that it was not only beneficial to the patients to be actively involved but that it also saved the institution added expense.

## The chaplain and the superintendent

Music, literature and education were all part of the recreational aspect of moral treatment; an element, which often demanded and valued collaboration between patients, staff and the wider public. Superintendents and chaplains were two of the most senior officers of the asylum, and it was thus important for them to establish a good working relationship. However, apart from occasional personal differences, tensions could also arise from the potential conflation of the religious and the medical sphere.^133^

As far as hymn and prayer books were concerned, there were many different types to choose from, and one particular example were consolation texts for the invalid reader.^134^ In these, as Maria Frawley suggested, “is little or no reference to interaction with medical men” but “instead, God functions as the source of understanding and comfort.”^135^ There are no titles of hymn or prayer books listed in the primary asylum literature consulted, but as “many dozens of spiritual texts devoted to the enrichment of the Christian invalid's character (…) poured forth from religious and independent presses and agencies in the nineteenth century,” addressing mental illnesses such as hypochondria, it is possible that these types of publications formed part of the reading material offered by asylum chaplains.^136^ Frawley thus concluded that “if Evangelicalism helped to usher the physician out of the sickroom, the medical profession might just as easily intervene to usurp diagnostic control of the Christian sufferer.”^137^

It is exactly this connection between the medical and the religious sphere which had the potential to create friction between asylum chaplains and superintendents. Several physicians and clergymen engaged with this issue. At the height of the reform movement in the 1840s, a time when alienists were trying to establish themselves as recognised specialists in the field of insanity, physician Nathanial Bingham had proposed in his assessment of the asylum chaplain's position that he should exercise his duties “subject to the approval of the medical officer” whose “post is one of command,” and “now and then be mediator between them [the patients] and the superintendent.”^138^ Along similar lines, physician Arthur Ladbroke Wigan upheld that “If the office of medical adviser and spiritual guide were united in one person, (…) the task of managing and of curing insanity, when curable, would be comparatively easy,” yet “in the management of the insane, the sincere co-operation of the two is essentially necessary.”^139^

However, voices in favor of placing the insane under the care of religious orders became louder. Often these referred to practices on the continent, for example, as described in a contribution to the *Journal of Mental Science* in 1868. After outlining the history of religious communities establishing and running asylums in France, the author came to the conclusion that they “can be best worked by religious [*sic*] or those trained by them.”^140^ Another article cited German physicians, concluding that “if insanity is not a disease of the brain, but of the soul, why should not deference be paid to the opinions of the clergy” and thus, “it appears (…) indisputable, that in the domain of what is called Psychology, it is not the clergy who are intruders but the medical men themselves.”^141^ The writer, however, then seemed to distance himself from this view by rebutting that at least “in England, insanity is held to be as purely a disease of the brain,” implying therefore that the previous argument was disarmed.

Perhaps the greatest controversy was caused by the anonymous author of a book entitled *The Priest in Absolution.* He proclaimed that “as the Priest should have impressed upon him the character of a father, so should he add to this the skill of a physician,” as he embodied “Christ, the Good Physician, healing the sick.”^142^ This work alarmed the higher echelons of the Anglican clergy as it was seen as a threat to the Established Churches, but it also prompted opposition from physicians.^143^ J.C. Bucknill, for example, acknowledged “that a certain school or sect of divines within the State Church of this country are diligently employed in the dissemination of the doctrine that the priest is a physician.”^144^ His main concern, however, was the damage it would do to the reputation of the alienist. He concluded that “if we allow this doctrine to pass unquestioned, there may perhaps be little danger that priests will really seek to practice as physicians; but there is a likelihood that the public estimate of the functions of the physician will be unsettled and confused.”^145^

This doctrine did *not* pass unquestioned. An article in the *Journal of Mental Science* outlined the role of the chaplain and the attributes he should possess. Within this, the author asserted that “another fundamental axiom with the Good Chaplain is the duty of loyalty to the Medical Superintendent” and that “he realizeth that their common object is the welfare and encouragement of the patient, and that although they approach man's dual nature from different standpoints, there is no occasion to clash.”^146^ Another article in the same journal referred to opposition even on the continent, where the editor of the *Zeitschrift für Psychiatrie*, H. Laehr, posited that the “physician must take the first place in dealing with the insane, and must have the direction of the treatment.”^147^

## The chaplain and his patients

John Conolly referred to this particular interplay between the medical and the religious sphere, believing “that many insane persons are capable of deriving much satisfaction from being permitted to attend services (…) and that a good and prudent clergyman may become a useful auxiliary to a physician, by correcting fanatical delusions, moderating spiritual conceit.”^148^ However, if the onus of making religious activities a success for patients was thus placed in the hands of the chaplain, the selection of those taking part in them rested predominantly with the superintendents. A statement by Reverend Pulling exemplifies this. He described how he “had conversed with all those who have been disposed to talk with me, and whom I have considered likely to derive some benefit from my so doing,” adding that he had “not been forbidden by the superintendent to visit any of the Patients.”^149^ This confirmed that, at Littlemore at least, the ultimate decision lay with the medical man in charge. Yet exactly what type of patient was deemed the most suitable to attend religious activities? Again, no real consensus was found. Browne suggested that “upon the discrimination of the patients to whom religious instruction is adapted, the whole question of its utility rests.”^150^ This, invariably, was subjective. Patients not selected seemed to feel this exclusion keenly. Henry Murray reported that he was often approached in the refractory wards by patients begging him to be allowed to attend Chapel, and how it grieved him to decline this. He cites one particular patient who, after his recovery and discharge, told of his distress at having been denied access.^151^

Of course, it is, as Louise Hide has suggested with regards to patients at Claybury Asylum, perhaps “likely that many were indifferent to religion and chapel.”^152^ The fact that John Conolly told attendants to “take care that every patient desirous of going to the chapel should be ready to go when the bell rings; and they should remind some, who take little notice of these preparations, that if they wish it they can also attend,” confirms this.^153^ However, there is evidence that many patients very much appreciated participating in services. Henry Hawkins recalled a letter from a recovered, discharged patient, who stated that “the services at the chapel which I have had the privilege of attending have been to me, as it were, wayside seats, where I have often sat and refreshed myself.” ^154^ At Littlemore, a former female patient, who had also been part of the reading group, told the chaplain that “when she was in a most distressed and lost state of mind she found great comfort from the words of one of the Psalms.”^155^

Other discharged patients explained how it “enabled them to bear their affliction, and temporary ‘confinement’ with much greater patience,” and how it offered “support and comfort for the remainder of the week”, leading the chaplain to believe that “the memory of the many insane persons is surprisingly retentive.”^156^ Thomas Downie recalled that frequently “patients who had recovered from their illness, and were about to leave the institution, expressed to me their gratitude for the benefit they had derived from the religious services held in it.” He then presented an extract from a letter written to him by “a gentleman of high intelligence and culture, who had been for some time in the asylum, but had recovered”:

I cannot close (…) without expressing the pleasure I had, during my stay at Morningside, especially in the Sabbath services, which were to me like streams in the desert, or rather, I should say, like wells of living water. I am much impressed with the importance of your sphere there, and the noble opportunity of serving the Master in a position requiring much tact and wisdom. I know that not a few there are looking to you for spiritual advice.^157^

The reliability of patient testimony and potential biases must of course be considered. Allan Beveridge explored the various reasons behind the many letters analysed at Morningside, pointing out that often the content of a letter was used to confirm the level of insanity of a patient, and that patients who wrote back to the asylum after their release were also more likely to report a positive experience than those remaining institutionalized.^158^ Nonetheless, the examples cited above are expressing patient opinions which should be heard and which give a clear indication that, as well as the other activities facilitated by the chaplain, religious services were enjoyed and deemed therapeutic by some of the asylum congregation. It thus complicates the picture of the asylum as a “museum of madness” where patients had little agency and even less joy. It also offers an opportunity to reconsider the role religion, spirituality and the clergy could potentially play in the treatment and prevention of mental ill health today.

## Conclusion

As sociologist Wendy Cadge has observed, the role of today's faith-based chaplains is largely unknown and under-appreciated.^159^ This is also true of the 19th-century clergymen who worked, and often lived, in institutions for the insane. This article has sought to rectify this and show how crucial they were in the day-to-day lives of patients, as their duties straddled the religious, recreational and medical spheres. It has provided a historical perspective which perhaps can also raise the profile of their modern day counterparts and lend credence to the current research by, amongst others, psychologist David Rosmarin and psychiatrist Harold Koenig, which demonstrates that spirituality and religion are “strongly tied in both positive and negative ways to (…) mental health” and that it “can clearly inform our understanding of the cause and aetiology of mental disorders and also enhance methods of clinical treatment.”^160^

As part of moral treatment, religion played a central role in 19th-century asylums and was imbued with managerial as well as therapeutic potential. Chaplains had to offer regular services which were not too dissimilar to those provided in local parishes. However, to counteract concerns about religion causing or exacerbating religious insanity, and to cater for a congregation with different medical needs, chaplains had to adapt services in language and length. As the 19th century progressed, they were faced with the challenges of overcrowded institutions and often chronically ill patients, rendering individual pastoral care more difficult; chaplains acknowledged this in their reports, yet continued with their therapeutic missions lifted “by the gratitude” extended to them by many of their flock.^161^

Attendance figures and patient testimony confirm the popularity and positive impact of religious services and spiritual aid, yet what makes the 19th-century asylum chaplain stand out is his involvement in the recreational life of the institution and its patients. As moral treatment gained traction, he further “diverted their thoughts from themselves” by overseeing the library, keeping an eye on the musical developments in chapel, as well as arranging lectures, reading and writing classes and “innocent amusements” such as Magic Lantern shows. Challenging our perception of asylums as places of repression and neglect where patients had very little agency, his ministry was at once consoling, entertaining, educational, and, in consequence, deemed therapeutic. This had the potential to create tensions between him and the physician, but, in the end, cooperation prevailed. Therefore, so this article proposes, chaplains ought to be recognized in the asylum historiography as being instrumental in the implementation of moral treatment; not just because of their role as providers of religious services but also for their involvement in some of the asylum recreations in collaboration with other staff.

We also need to acknowledge that the chaplain's role was not an easy one; his congregation was challenging, and he was often isolated and stigmatized. His position was fixed as were his duties, but their extent was frequently dependent on the enthusiasm and dedication of the individual. Many chaplains, such as Henry Higgins, Edwin Pulling or Henry Hawkins were exemplary and longstanding, and this article pays a long overdue tribute to their achievements. As the anonymous reviewer of Hawkins’ publications asserted:

As in everything else, so does it hold good, unfortunately, in the sphere of asylums for the insane—there are chaplains and chaplains; those who hold their office for no higher purpose than to make a livelihood, and those who, like Mr. Hawkins, perform their duties as a labour of love.^162^

## Footnotes

1 Thomas Downie, “Religion, and its Influence upon the Insane,” *Journal of Mental Science* (henceforth *JMS*) 39 (1893): 558–59, 558.

2 On the role of religion see for example Leonard D. Smith, “*Cure, Comfort and Safe Custody”: Public Lunatic Asylums in Early Nineteenth-Century England* (London/NY: Leicester University Press, 1999), 209–11; if at all, chaplains usually get mentioned only briefly and are very rarely named, for example in Catherine Cox, Hilary Marland, Sarah York, “Emaciated, Exhausted, and Excited: The Bodies and Minds of the Irish in Late Nineteenth-Century Lancashire Asylums,” *Journal of Social History* 46 (2012): 500–52, 515, or Anna Shepherd, *Institutionalizing the Insane in Nineteenth-Century England* (London: Pickering & Chatto, 2014), 63; Rosemary Golding, “Sounding the Archival Silence: Searching for Music in the Nineteenth-Century English Asylum,” *Social History of Medicine* 35 (2022): 1223–46, 1233.

3 For example, Andrew Scull, “Mad-Doctors and Magistrates: English Psychiatry's Struggle for Professional Autonomy in the Nineteenth Century,” *European Journal of Sociology* 17 (1976): 279–305; Leonard D. Smith, “Behind Closed Doors; Lunatic Asylum Keepers, 1800–60,” *Social History of Medicine* 1 (1988): 301–27; David Wright, “The Dregs of Society? Occupational Patterns of Male Asylum Attendants in Victorian England,” *International History of Nursing Journal* 1 (1996): 5–19.

4 For example P. T. Syrée, “The Asylum Chaplain ‘Ministering to the Mind Diseased’,” *JMS* 40 (1894): 252–53; Anon., “The Good Asylum Chaplain,” *JMS* 39 (1893): 399–401; Henry Hawkins, “‘Ut Co-operatores simus’: A Plea for Daily Services in Asylum Chapels,” *JMS* 40 (1894): 424–26; Edward G. O'Donoghue, *The Story of Bethlehem Hospital from its Foundation in 1247* (London: T. Fisher Unwin, 1914).

5 Wellcome Library Online (henceforth WLO), *Report from Select Committee on Pauper Lunatics in the County of Middlesex, and on Lunatic Asylums,* 1827, Appendix 3, 11; Jane Freebody, *Work and Occupation in French and English Mental Hospitals, c.1918–1939* (Cham: Palgrave Macmillan, 2023); Ute Oswald, “‘Distraction from Hurtful Thoughts’: Recreational Activities as Agents of Healing in Nineteenth-Century British Asylums,” *Medizinhistorisches Journal* 56 (2021): 30–57.

6 Andrew Scull, “Moral Treatment Reconsidered: Some Sociological Comments on an Episode in the History of British Psychiatry,” *Psychological Medicine* 9 (1979): 421–28; Anne Digby, “Moral Treatment at the Retreat, 1796–1846.” In *The Anatomy of Madness: Essays in the History of Psychiatry II: Institutions and Society*, edited by W. F. Bynum, R. Porter and M. Shepherd (London/New York: Tavistock, 1985), 52–72; Smith, “*Cure, Comfort and Safe Custody”*, 187–94. On sport see Rob Ellis, “Asylums and Sport: Participation, Isolation and the Role of Cricket in the Treatment of the Insane,” *The International Journal of the History of Sport* 30 (2013): 83–101; Steven Cherry and Roger Munting, “‘Exercise is the Thing?’ Sport and the Asylum 1850–1950,” *The International Journal of the History of Sport* 22 (2005): 42–58; on theatre see Juliet Foster, “Performance in Bethlem, Fulbourn and Brookwood Hospitals: A Social Psychological and Social Historical Examination.” In *Madness, Performance, Psychiatry: Isolated Acts*, edited by Anna Harpin and Juliet Foster (Basingstoke: Palgrave, 2014), 47–50; on music see Rosemary Golding, *Music and Moral Management in the Nineteenth-Century English Lunatic Asylum* (Cham: Palgrave Macmillan, 2021); Golding, “Sounding the Archival Silence.”

7 Apart from Golding, *Music and Moral Management*, 76–79, who briefly describes music in religious practice, yet here without specific mention of chaplains.

8 Andrew Scull, *The Most Solitary of Afflictions: Madness and Society in Britain, 1700–1900* (New Haven, CT/London: Yale University Press, 1993), 289.

9 O. Stavrova, D. Fetchenhauer and T. Schlösser, “Why Are Religious People Happy? The Effect of the Social Norm of Religiosity Across Countries,” *Social Science Research* 42 (2013): 90–105.

10 Paul Summergrad, “Foreword.” In *Handbook of Spirituality, Religion, and Mental Health*, edited by David H. Rosmarin and Harold G. Koenig (Cambridge, MA: Academic Press, 2020), xiii–xv.

11 Leonard D. Smith, “Insanity and Ethnicity: Jews in the Mid-Victorian Lunatic Asylum,” *Jewish Culture and History* 1 (1998): 27–40; Rob Ellis, *London and its Asylums, 1888–1914: Politics and Madness* (London: Palgrave Macmillan, 2020), 211–15.

12 Smith, “Insanity and Ethnicity”, 36–37; see also WLO, 45th Report of the Commissioners in Lunacy, 1890, 198 on the visitations of a rabbi; on Catholic services see WLO, Reports of the County Lunatic Asylums at Lancaster, Prestwich, Rainhill, and Whittingham 1874, 59, 164, 171.

13 For a representative example, at the Moulsford Lunatic Asylum in Berkshire, out of 100 patients admitted in 1883, 74 were Church of England, with the remaining 26 scattered across 10 other denominations, including 6 Baptists, 1 Catholic and 2 Congregationalists. WLO, Moulsford Lunatic Asylum, Annual Report (henceforth AR), 1883, 39.

14 Jonathan Andrews *et al*., *The History of Bethlem* (London: Routledge, 2013), 25–26; 80–82, 113; see also O'Donoghue, *The Story of Bethlehem Hospital,* 363–69.

15 Digby, “Moral Treatment at the Retreat,” 53.

16
*Ibid.*

17 Smith, “*Cure, Comfort and Safe Custody*,” 209.

18 Gerald Parsons, “Introduction: Victorian Religion, Paradox and Variety.” In *Religion in Victorian Britain I: Traditions*, edited by Gerald Parsons (Manchester/New York: Manchester University Press, 1988), 8.

19 Julie Melnyk, *Victorian Religion: Faith and Life in Britain* (Westport, CT/London: Praeger, 2008), 13.

20 Dominic Erdozain, *The Problem of Pleasure: Sport, Recreation and the Crisis of Victorian Religion* (Woodbridge: Boydell Press, 2010), 4; for a detailed discussion of different denominations see Melnyk, *Victorian Religion,* 19–64.

21 Melnyk, *Victorian Religion*, 86.

22 Geoffrey B. A. M. Finlayson, *The Seventh Earl of Shaftesbury* (Vancouver: Regent College Publishing, 2004), 153–71; Brian Harrison, “Religion and Recreation in Nineteenth-Century England,” *Past & Present* 38 (1967): 98–125, 99.

23 House of Lords, 9 Geo. IV, c. 40, Amended County Asylums Act of 1828, Bill number 111, 2 June 1828, 17.

24 House of Lords, *A Bill [as Amended in Committee] Intituled “An Act to amend the Laws for the Provision and Regulation of Lunatic Asylums for Counties and Boroughs, and for the Maintenance and Care of Pauper Lunatics, in England,”* Bill number 848, 29 July 1845, 26–27.

25 WLO, Report of the Commissioners in Lunacy 1847; WLO, Report of the Commissioners in Lunacy 1859; WLO, Report of the Commissioners in Lunacy 1890.

26 For example, at Hatton Asylum, Warwick, Warwickshire County Record Office, Chaplain's Journal, CR3162/105 and at Moulsford Lunatic Asylum, Berkshire Record Office, Chaplain's Journals D/H10/E1.

27 Kathleen Jones, *Asylums and After: A Revised History of the Mental Health Services: From the Early 18^th^ Century to the 1900s* (London/New Brunswick, NJ: Athlone Press, 1993), 116.

28 Scull, *The Most Solitary of Afflictions,* 281.

29 1829 at Hanwell and 2093 at Colney Hatch: WLO, Hanwell Asylum (henceforth HA), AR 1875, 3; WLO, Colney Hatch Asylum, AR 1875, 15; Oxford History Centre (henceforth OHC), OHA L1/A1/2/3, Littlemore Asylum, Report of the Committee of Visitors 1874, 7-8.

30 WLO, Report of the Commissioners in Lunacy 1859, 37-38.

31 London Metropolitan Archives (henceforth LMA), H11/HLL/A/7/1, HA, AR 1839, 27; staff regularly took part in services, or even had their own, see Louise Hide, *Gender and Class in English Asylums, 1890–1914* (Basingstoke: Palgrave Macmillan, 2014), 119-20.

32 LMA, H11/HLL/A/5/2, HA, Chaplain's Report, 1842, 51; WLO, The Reports of the Visiting Justices of the County Lunatic Asylum at Hanwell, from the Easter Sessions 1839 to the Michaelmas Sessions, 1842, 221, 239; John Conolly, *The Construction and Government of Lunatic Asylums and Hospitals for the Insane* (London: John Churchill, 1847), 89.

33 LMA, H11/HLL/A/5/4, HA, Chaplain's Report 1855, 25.

34 LMA, H11/HLL/A/5/5, HA, AR, App. 1, Visiting Book of the Asylum, 32.

35 LMA, H11/HLL/A/5/13, HA, Chaplain's Report 1886, 36.

36 WLO, Surrey County Lunatic Asylum, Brookwood, AR 1870, 50.

37 WLO, Worcester County Lunatic Asylum, AR 1898, 10.

38 Melnyk, *Victorian Religion,* 66.

39 Hawkins, “‘Ut Co-operatores simus’,” 425.

40 Smith, “*Cure, Comfort and Safe Custody*,” 210.

41
*Lancaster Gazette,* 28 October 1826, 3.

42 LMA, H11/HLL/A/7/1, HA, AR 1839, 27; Conolly, *Construction and Government,* 126.

43 Michael MacDonald, *Mystical Bedlam: Madness, Anxiety and Healing in Seventeenth-Century England* (Cambridge: Cambridge University Press, 1983), 206.

44 Robert Burton, *The Anatomy of Melancholy*, Holbrook Jackson, ed. (New York: New York Review of Books, 2001), 364; Roy Porter, *Mind Forg’d Manacles: A History of Madness in England from the Restoration to the Regency* (London: Penguin, 1990), 264–68.

45 Roy Porter, *A Social History of Madness* (New York: Weidenfeld & Nicholson, 1987), 93; on Wesley's visits to Bethlem, his ban and the ‘outbursts of hysteria’ see O’Donoghue, *The Story of Bethlehem Hospital*, 255; James Cowles Prichard, *A Treatise on Insanity and Other Disorders Affecting the Mind* (London: Sherwood, Gilbert and Piper, 1835), 188.

46 WLO, Devon County Lunatic Asylum, AR 1874, 33; WLO, HA, AR 1874, 96.

47 John Conolly, “On the Physiognomy of Insanity, No. 9: Religious Mania,” *Medical Times and Gazette* 17 (1858): 81–83, 82; John Conolly, “On the Physiognomy of Insanity, No. 10: Religious Mania – Convalescence,” *Medical Times and Gazette* 17 (1858): 210-12; Daniel Hack Tuke, *Dictionary of Psychological Medicine,* (Philadelphia: Blakiston and Son, 1892), 765, 797, 1092, 1334–35.

48 For example, *Evening Star,* Monday 29 November 1886, 4; *Hull Daily News*, Saturday 25 September 1869, 5.

49 O’Donoghue, *The Story of Bethlehem Hospital*, 365.

50
*Bolton Evening News,* 30 May 1876, 4. On this debate see also *Nottingham Review and General Advertiser for the Midland Counties,* 15 November 1850, 5.

51 Anon., “The Good Asylum Chaplain,” *JMS* 39 (1893): 400.

52
*Ibid.,* 400-401.

53 OHC, OHA L1/A1/2/1, Littlemore Asylum, Chaplain's Report 1860, 20.

54 OHC, OHA WP80 1846, Warneford Asylum, AR, 1846, no page numbers; OHC, OHA L1/A1/2/3, Littlemore Asylum, Chaplain's Report, 1871, 26; LMA, H11/HLL/A/5/4, HA, Chaplain's Report 1854, 117; W. A. F. Browne, *What Asylums Were, Are, and Ought to Be* (London: Adam and Charles Black,1837), 211.

55 WLO, Colney Hatch Asylum, Chaplain's Report 1854, 117; LMA, H11/HLL/A/5/2, HA, Chaplain's Report 1842, 56.

56
*Lancaster Gazette*, 28 October 1826, 3; L. D. Smith, “A Gentleman's Mad-Doctor in Georgian England: Edward Long Fox and Brislington House,” *History of Psychiatry* 19 (2008): 163-84, 179.

57 WLO, Wandsworth Asylum, AR 1855, 27; LMA, H11/HLL/A/7/1, HA, AR 1836, 26. Very rarely do we read of any disturbances in chapel, for example due to epileptic fits or ‘excitement’; see WLO, Littlemore Asylum, Report of the Committee of Visitors 1853, 11. However, the selection process may have safeguarded against this, or perhaps such incidences were deliberately excluded.

58 On the social control debate see e.g. Scull, “Moral Treatment Reconsidered,” 421–28; Mark S. Micale and Roy Porter, eds, *Discovering the History of Psychiatry* (Oxford: Oxford University Press, 1994); Joseph Melling and Bill Forsythe, *The Politics of Madness: The State, Insanity and Society in England, 1845–1914* (London/New York: Routledge, 2006), 1–7.

59 Browne, *What Asylums Were, Are, and Ought to Be*, 211.

60 WLO, Report of the Commissioners in Lunacy to the Lord Chancellor, 1859, 18; WLO, Report from Select Committee on Pauper Lunatics in the County of Middlesex, and on Lunatic Asylums, 1827, Appendix 3, Minutes of Evidence, 17.

61 Browne, *What Asylums Were, Are, and Ought to Be*, 211.

62 WLO, Littlemore Asylum, AR, 1847, 8.

63
*Ibid.*

64 Downie, “Religion and its Influence on the Insane,” 558; Nathaniel Bingham, *Observations on the Religious Delusions of the Insane* (London: J Hatchard and Son, 1841), 166–167.

65 LMA, H11/HLL/A/5/7, HA, *Regulations and Orders for the Management of the Middlesex County Lunatic Asylum at Hanwell London* (1869), 46–47.

66 Shepherd, *Institutionalizing the Insane*, 63.

67
*Ibid.*

68 Syrée, “The Asylum Chaplain,” 252–53; Anon, “Priest and Physician,” *JMS* 44 (1898): 835.

69 Syrée, “The Asylum Chaplain,” 252–53; O’Donoghue, *The Story of Bethlehem Hospital*, 367.

70 O’Donoghue, *The Story of Bethlehem Hospital*, 367.

71
*Ibid.;* for example, Henry Hawkins regularly referred to correspondence between himself and friends of patients, see WLO, Colney Hatch Asylum, AR 1869, 82.

72 O’Donoghue, *The Story of Bethlehem Hospital*, 366–67.

73 WLO, Wandsworth Asylum, AR 1850, 20–21.

74
*Ibid*., 21; furthermore, at Hanwell, in 1874 a third Sunday service was added to accommodate the growing congregation, and the chaplain also “compensated by an increased payment.” WLO, HA, AR 1875, 9.

75 Syrée, “The Asylum Chaplain,” 253.

76 WLO, Cornwall County Lunatic Asylum, AR 1869, 11; Conolly, *Construction and Government,* 89; one example of a residency was at Whittingham, the fourth Lancashire County Asylum, where a special “Chaplain's House” was to be built in 1874; WLO, Reports of the County Lunatic Asylums at Lancaster, Prestwich, Rainhill, and Whittingham, 1874, 161.

77 OHC, OHA L1/A1/2/1, Littlemore Asylum, AR 1855, 18.

78 For example, at the Moulsford Asylum in 1883 the chaplain mentions three colleagues, one of which had preached the sermon at the Harvest festival's Evening Service. WLO, Moulsford Asylum, AR 1883, 46. The chaplain at the Warwick County Asylum, Hatton, noted in his 1890 journal that “owing to illness,” he had provided “substitutes.” Hatton Asylum, Warwick, Warwickshire County Record Office, *Chaplain's Journal* (1890), CR3162/105.

79 H. H., “The Asylum Chaplain's Column,” *JMS* 39 (1893): 410.

80 Anon, “Review of H. Hawkins, An Address to Asylum Attendants ‘Off Duty’-‘Invalided’,” *JMS* 33 (1888): 599–600, 599.

81 Vicky Long, *Destigmatising Mental Illness?: Professional Politics and Public Education in Britain, 1870–1970* (Manchester/New York: Manchester University Press, 2014), 168. From 1894 onwards this was made available also to male patients.

82 WLO, Reports of the County Lunatic Asylums at Lancaster, Prestwich, Rainhill, and Whittingham: Rainhill Asylum, AR, 1855, 95.

83 T. L. R., “The Asylum Chaplain's Column,” *JMS* 40 (1894): 66–67.

84 OHC, OHA L1/A1/2/1, Littlemore Asylum, AR, 1859, 19.

85
*Ibid.*

86
*Ibid.,* AR 1860, 19.

87 OHC, OHA L1/A1/2/2, Littlemore Asylum, AR, 1865, 37.

88
*Ibid*., AR 1863, 24.

89 David Bebbington, “The Growth of Voluntary Religion.” In *The Cambridge History of Christianity: World Christianities 1815–1914,* vol. 8, edited by Sheridan Gilley and Brian Stanley (Cambridge: Cambridge University Press, 2006), 57, 59; Erdozain, *The Problem of Pleasure*, 77, 124–25, 130; Parsons, “Introduction: Victorian Religion, Paradox and Variety,” 1–13, 8; Hugh McLeod, *Religion and Society in England, 1850–1914* (Basingstoke/London: Macmillan Press, 1996), 78–82.

90 Peter Bailey, *Leisure and Class in Victorian England: Rational Recreation and the Contest for Control, 1830–1885* (London: Methuen &Co, 1978), 116–17; Akihito Suzuki, “The Politics and Ideology of Non-Restraint: The Case of the Hanwell Asylum,” *Medical History* 39 (1995): 1–17, 13.

91 Peter Bailey, *Leisure and Class,* 83; Erdozain, *Problem of Pleasure,* 97.

92 Hugh McLeod, *Religion and the Working Class in Nineteenth-Century Britain* (Basingstoke/London: Palgrave Macmillan, 1984), 15, 40–41.

93 J. C. Bucknill and D. H. Tuke, *A Manual of Psychological Medicine* (Philadelphia: Blanchard and Lea, 1858), 499–500; W. A. F Browne, *The Moral Treatment of the Insane: A Lecture* (London: J.E. Adlard, 1864), 23; John Todd and Lawrence Ashworth, “The West Riding Asylum and James Crichton-Browne, 1818–76.” In *150 Years of Psychiatry*, edited by G. E. Berrios and H. L. Freeman (London: Gaskell, 1991), 386–418, 406–407.

94 Henry Hawkins, “Church Ministrations in Public Asylums,” *JMS* 16 (1870): 151–52, 151.

95 “Henry Higgins,” *Biographies.net.* STANDS4 LLC, 2025. Web. 8 Feb. 2025. <https://www.biographies.net/biography/henry-higgins/m/0h54qv8>.

96 OHC, OHA L1/A1/2/1, Annual Reports of Littlemore Asylum 1847–1861, AR 1859, 19; OHC, OHA L1/A1/2/2, Littlemore Asylum, AR 1866, 22; OHC, OHA L1/A1/2/3, Littlemore Asylum, AR 1875, 33.

97 For example, LMA, H11/HLL/A/7/1, HA, AR 1839, 28; LMA, H11/HLL/A/5/1, HA, AR 1840, 16.

98
*Ibid*.

99 LMA, H11/HLL/A/5/2, Annual Reports, HA, 1842–1843, Chaplain's Report 1842, 55.

100
*Ibid*.

101
*Ibid*., 55–56.

102
*Ibid.,* 56.

103
*Ibid*., AR 1843, 5.

104
*Ibid*.: a substantial number of patients took part throughout the year, viz 115 male and 65 female patients, resulting in five male classes and four female classes per day on average.

105 Conolly, *Construction and Government*, 129–30.

106 Noteworthy in this context is the connection with prison chaplains, who were also involved with education in their institutional practice, in particular J.T. Burt, who moved from Hanwell to Pentonville Prison. See Catherine Cox and Hilary Marland, *Disorder Contained, Mental Breakdown and the Modern Prison in England and Ireland, 1840–1900* (Cambridge: Cambridge University Press, 2022), 33–34, 49, 52, 61–69, 214; Burt later became chaplain at Broadmoor, the Criminal Lunatic Asylum, and supervised classes there too: WLO, Broadmoor Criminal Lunatic Asylum, AR 1869, 10.

107 Hide, *Gender and Class*, 120.

108 OHC, OHA L1/A1/2/1, Littlemore Asylum, AR 1847, 7; *Ibid*., AR 1850, 11.

109
*Ibid,* AR 1855, 17.

110 Judith Flanders, *Consuming Passions: Leisure and Pleasure in Victorian Britain* (London: Harper Press, 2006), 184–85; Bailey, *Leisure and Class in Victorian England,* 72, 86; on Penny Readings, where the working man was “lectured to, or read to, by his brother in purple and fine linen” see Hugh Cunningham, *Leisure in the Industrial Revolution, c. 1780–1880* (London/New York: Routledge, 1980), 123.

111 Pamela Horn, *Amusing the Victorians: Leisure, Pleasure and Play in Victorian Britain* (Stroud: Amberley, 2014), 303–304.

112 WLO, HA, AR 1843, 57.

113 LMA, H11/HLL/A/5/4, HA, AR 1857, 78.

114 LMA, H11/HLL/A/5/7, HA, AR 1871, 28; on the more general role of reading in asylums see Laura Blair, “‘Library as Laboratory’: Moral Treatment, Patient Libraries and Reading in Nineteenth-Century British Asylums” (unpublished PhD thesis, Queen Mary University of London, 2024).

115 Parsons, “Victorian Religion, Paradox and Variety,” 6–7.

116 Melnyk, *Victorian Religion*, 78–79.

117 LMA, H11/HLL/A/5/4, HA, AR 1857, 78; LMA, H11/HLL/A/5/5, HA, AR 1863, 47; LMA, H11/HLL/A/5/8, HA, AR 1872, 42.

118 OHC, OHA L1/A1/2/1, Littlemore Asylum, AR 1855, 17–18.

119 David Uwins, *A Treatise on Those Disorders of the Brain and Nervous System, Which Are Usually Considered and Called Mental* (London: Renshaw and Rush, 1833*)*, 184. See also Hawkins, “Ut Co-operatores simus,” 425 on wider benefits of religious activities for staff.

120 OHC, OHA L1/A1/2/1, Littlemore Asylum, Chaplain's Report 1855, 15.

121 Downie, “Religion and Its Influence on the Insane,” 559; Arthur Ladbroke Wigan, *A New View of Insanity: The Duality of the Mind Proved by the Structure, Functions, and Disease of the Brain* (London: Longman et al., 1844), 428.

122 Golding, “Sounding the Archival Silence,” 1233.

123 WLO, Colney Hatch Asylum, Chaplain's Report, 1854, 118.

124 Melnyk, *Victorian Religion*, 20; 36; 69.

125 Hawkins, “Ut Co-operatores simus,” 426; H. H., “The Asylum Chaplain's Column,” 411; Hawkins, *History of Colney Hatch,* 16 on the choir at Colney Hatch; on the participants in religious music in asylums see also Golding, *Music and Moral Management,* 77–78.

126 H. H., “The Asylum Chaplain's Column,” 411.

127 WLO, Devon County Lunatic Asylum, AR 1872, 21; WLO, Devon County Lunatic Asylum, AR 1874, 21.

128 OHC, OHA WP80 1874, Warneford Asylum, AR 1874, 9; OHC, OHA WP80 1872, Warneford Asylum, AR 1872, 6; WLO, Dorset County Lunatic Asylums, Charminster and Forston, AR 1873, 14.

129 Also when expressing a wish to receive one: for example, at Derby County Lunatic Asylum: WLO, Report of the Commissioners in Lunacy 1871, 144.

130 LMA, H11/HLL/A/7/1, HA, AR 1839, 37.

131 WLO, Devon County Lunatic Asylum, AR 1872, 21; OHC, OHA L1/A1/2/2, Littlemore Asylum, AR 1865, 34.

132 H11/HLL/A/5/3B, Colney Hatch Asylum, Chaplain's Report 1853, 118.

133 On the personal tensions between John Conolly and Rev. Tebbutt for example, see Suzuki, “Politics and Ideology,” 9, fn50; on the loyalty between Rev. Burt and Conolly see *ibid*., 16.

134 Melnyk, *Victorian Religion*, 70–71.

135 Maria H. Frawley, *Invalidism and Identity in Nineteenth-Century Britain* (Chicago: Chicago University Press, 2004), 163.

136
*Ibid*., 73 (on hypochondria), 158; unless perhaps they were deemed to be unsuitable.

137
*Ibid.,* 167.

138 Bingham, *Observations on the Religious Delusions of Insane Persons,* 165–66.

139 Ladbroke Wigan, *A New View of Insanity*, 430–31.

140 J. R. G., “Notes on the French Asylums for the Insane Under the Care of Religious,” *JMS* 14 (1868): 288–91, 291.

141 Anon., “Religious Societies and Clerical Influences in Asylums, from the Allgemeine Zeitschrift für Psychiatrie und Psychischgerichtliche Medicine, 12ter Band, 2tes Heft,” *Asylum Journal of Mental Science* 2 (1855): 111–13, 113.

142 Anon., *The Priest in Absolution*, *A Manual for Such as Are Called unto the Higher Ministries in the English Church* (London: Joseph Masters, 1866), 5, 46.

143 The Confessional- “The Priest in Absolution”- Observations, *Hansard*, House of Lords Debate 14 June 1877, vol 234, cc 1741-53.

144 Anon., “Dr. Bucknill on Specialism, Confession, and the Physician v. the Priest,” *JMS* 23 (1877): 362–68, 364.

145
*Ibid*.

146 Anon., “The Good Asylum Chaplain,” 400.

147 Anon., “Priest and Physician,” 835.

148 Conolly*, Construction and Government of Lunatic Asylums,* 124.

149 WLO, Littlemore Asylum, AR 1847, 8.

150
*Ibid*., 209.

151 WLO, Colney Hatch Asylum, Chaplain's Report 1854, 116–18.

152 Hide, *Gender and Class*, 120.

153 Conolly, *Construction and Government of Lunatic Asylums*, 124.

154 Hawkins, “Ut Co-operatores simus,” 424.

155 OHC, OHA L1/A1/2/1, Littlemore Asylum , AR 1860, 18.

156
*Ibid*.

157 Downie, “Religion and Its Influence on the Insane,” 559; for other letters from recovered patients to their asylum chaplains, see Hawkins, “Ut Co-operatores simus”, 424.

158 Allan Beveridge, “Life in the Asylum: Patients’ Letters from Morningside, 1873–1908,” *History of Psychiatry* 9 (1998): 431–69, 434–37.

159 Wendy Cadge, *Spiritual Care: The Everyday Work of Chaplains* (Oxford: Oxford University Press, 2023), 5.

160 Rosmarin and Koenig, *Handbook of Spirituality, Religion, and Mental Health*, xvii.

161 WLO, Devon County Lunatic Asylum, AR 1874, 23.

162 Anon, “Review of H. Hawkins,” 599.

